# Through the Eyes of the Viewer: The Cognitive Load of LLM-Generated vs. Professional Arabic Subtitles

**DOI:** 10.3390/jemr18040029

**Published:** 2025-07-14

**Authors:** Hussein Abu-Rayyash, Isabel Lacruz

**Affiliations:** Department of Modern and Classical Language Studies, Kent State University, Kent, OH 44240, USA

**Keywords:** audiovisual translation, cognitive load, eye tracking, Arabic subtitles, large language models, GPT-4o, machine translation, cross-script processing, subtitle automation, viewing behavior

## Abstract

As streaming platforms adopt artificial intelligence (AI)-powered subtitle systems to satisfy global demand for instant localization, the cognitive impact of these automated translations on viewers remains largely unexplored. This study used a web-based eye-tracking protocol to compare the cognitive load that GPT-4o-generated Arabic subtitles impose with that of professional human translations among 82 native Arabic speakers who viewed a 10 min episode (“Syria”) from the BBC comedy drama series State of the Union. Participants were randomly assigned to view the same episode with either professionally produced Arabic subtitles (Amazon Prime’s human translations) or machine-generated GPT-4o Arabic subtitles. In a between-subjects design, with English proficiency entered as a moderator, we collected fixation count, mean fixation duration, gaze distribution, and attention concentration (K-coefficient) as indices of cognitive processing. GPT-4o subtitles raised cognitive load on every metric; viewers produced 48% more fixations in the subtitle area, recorded 56% longer fixation durations, and spent 81.5% more time reading the automated subtitles than the professional subtitles. The subtitle area K-coefficient tripled (0.10 to 0.30), a shift from ambient scanning to focal processing. Viewers with advanced English proficiency showed the largest disruptions, which indicates that higher linguistic competence increases sensitivity to subtle translation shortcomings. These results challenge claims that large language models (LLMs) lighten viewer burden; despite fluent surface quality, GPT-4o subtitles demand far more cognitive resources than expert human subtitles and therefore reinforce the need for human oversight in audiovisual translation (AVT) and media accessibility.

## 1. Introduction

Streaming services and other digital outlets have sparked a worldwide hunger for video content and reshaped how audiences discover foreign-language audiovisual (AV) materials. Ref. [1] observes that viewers now expect translated versions as a matter of course, a pattern encouraged by the effortless reach and on-demand nature of modern media. The translation sector now faces intense pressure because the customary process of subtitle preparation over several weeks no longer matches viewers’ expectations of instant availability. Globalization forces translators to render movies in very little time, yet still meet high standards [2]. This tension between speed and quality has accelerated interest in automated methods, and recent large language models (LLMs) appear poised to redefine subtitle production.

Subtitling involves much more than putting dialog into another language. Ref. [3] defines it as “rendering in writing, usually at the bottom of the screen, the translation into a target language of the original dialog exchanges […] as well as all other verbal information that appears written onscreen (letters, banners, inserts) or is transmitted aurally in the soundtrack (song lyrics, voices off).” Viewers must juggle several streams of information at once within tight temporal limits. The task becomes harder when the subtitle language uses a script unlike that of the image track, as in the case of Arabic readers who view English-language AV materials. Arabic runs right-to-left and has distinctive letter shapes, traits that pose demands few automated systems anticipate. Ref. [4] found that “when Arabic was read, performance was superior when windows extended leftward […] essentially the reverse of […] English,” evidence that reading direction guides visual attention. Such asymmetry could alter how audiences split their gaze between text and picture.

The cognitive effects of LLM-driven subtitling systems remain uncertain. Earlier work on machine translation (MT) provides a foundation, yet LLMs such as GPT-4o represent a major expansion in capability, generating context-aware responses that mimic human language so closely that readers often cannot tell the difference [5], but that very fluency can conceal inaccuracies, inconsistencies, and other misleading or harmful content in their output. Ref. [6] examined automatic subtitles produced with speech-recognition tools and found they often appear too quickly and in verbatim form, which overwhelms viewers and harms comprehension. These findings hint that artificial intelligence (AI), now an important tool for automated translation and subtitle production, still struggles to balance accuracy with readability, a compromise that expert subtitlers achieve through practice.

This study tests whether Arabic subtitles generated by GPT-4o impose greater cognitive load than those prepared by professional translators. We employ eye tracking to capture direct behavioral markers of mental effort. Cognitive load is “a theoretical construct describing the internal processing of tasks that cannot be observed directly” [7]. To infer that load, we examine fixation counts, dwell times, and the proportion of visual attention allocated to subtitle text versus on-screen imagery. Earlier work shows that language proficiency strongly shapes subtitle processing [8], so we also ask whether knowledge of English moderates these effects. We hypothesize that GPT-4o subtitles will demand more cognitive resources, visible in higher fixation counts (more frequent refixations signaling local processing difficulty), longer dwell times (extended cumulative gaze indicating sustained decoding effort), and shifts in gaze between textual and visual elements (additional saccades required to reconcile subtitle meaning with visual context). Such effects may be most pronounced among viewers with advanced English proficiency, who can spot flaws in translation quality.

## 2. Literature Review

Research on subtitled media sits at the crossroads of translation studies, cognitive psychology, and media accessibility. Ref. [9] defines audiovisual translation (AVT) as “a branch of translation studies concerned with the transfer of multimodal and multimedial texts into another language and/or culture.” Insights from each discipline clarify how audiences handle multilingual video, an issue that grows more pressing as global media habits evolve and new technologies appear. To understand the cognitive impact of subtitle quality, especially now that automated systems play a larger role, scholars draw on decades of work that explains how the visual and cognitive systems cope with the split task that requires viewers both to read the subtitle line and to follow the moving image.

Subtitled video demands complex cognitive work and reshapes how audiences watch films. Ref. [10] used eye tracking and found that “the addition of captions to a video resulted in major changes in eye movement patterns, with the viewing process becoming primarily a reading process.” Their result shows that audiences do not passively receive subtitles; instead, they reorganize attention so print commands most visual focus and alters engagement with the narrative.

Researchers describe this shift as automatic and involuntary. Ref. [11] argues that readers treat subtitles as routine but Ref. [12] go further, stating that “reading a subtitle at its onset presentation is more or less obligatory.” Evidence since then has shown that viewers follow subtitles even when they know the spoken language, and this tendency barely changes with soundtrack availability or plot action. To explain this pattern, Ref. [12] proposed two hypotheses. The efficiency hypothesis holds that text offers faster access than speech because viewers can revisit a subtitle, whereas they cannot replay the soundtrack. The familiarity hypothesis suggests that repeated exposure turns subtitle use into habit. The efficiency hypothesis aligns with limited capacity theories of attention [13], while the familiarity hypothesis relates to automatization through repeated exposure [14]. Both concepts are fundamental to understanding automatic processing in cognitive psychology. Both explanations show how deeply subtitle processing embeds itself in experienced viewers.

Furthermore, to explain automatic processing and its consequences, scholars examine the cognitive architecture that supports multimedia comprehension. Cognitive load theory (CLT) is “concerned with relationships between working and long-term memory and the effects of those relationships on learning and problem solving” [15]. CLT separates mental effort into intrinsic load, which arises from task complexity; extraneous load, which stems from information presentation; and germane load, which builds knowledge structures [16]. In subtitling, extraneous load rises whenever poor timing or weak translation forces needless effort. A viewer must attend to soundtrack, on-screen text, and moving images while they juggle both the foreign and the native languages [17], and the duplication of meaning across channels demands regular checks that can trigger overload [18]. Yet audiences adapt. When subtitles follow accepted standards, viewers maintain comprehension of plot and visual detail without strain, but timing errors, lengthy lines, or mistranslations drain resources and disrupt that balance [19].

Eye-tracking research offers a window onto the cognitive costs of processing complex visuals, because where and how long people look serves as a proxy for covert attention and effort. Distribution-based indices, such as the proportion of fixation time across competing onscreen elements, reveal how viewers prioritize information when multiple sources vie for attention [18]. Building on well-established dual-process theories of attention, the K-coefficient offers a more sophisticated method of quantifying the distribution of visual attention. It was first presented by [20] and, to facilitate statistical analysis, it expresses data in standard deviation units. Its concept is rather obvious: “positive values of Ki show that relatively long fixations were followed by short saccade amplitudes, indicating focal processing [whereas] negative values of Ki refer to the situation when relatively short fixations were followed by relatively long saccades, suggesting ambient processing” [20]. This dynamic approach contrasts with alternative classification schemes that rely on coarser gaze metrics; for example, Ref. [21] distinguished “overview” and “focused” viewing by counting transitions between predefined areas of interest, a technique difficult to generalize to studies lacking area-of-interest-level analyses. The K-coefficient’s ability to track real-time fluctuations in cognitive effort makes it particularly valuable for subtitle research, where higher K values indicate viewers moving from casual text skimming to concentrated reading and deeper processing, a shift that resembles cognitive load theory’s distinction between automatic and controlled processing during demanding multimodal activities.

Understanding cognitive processing has become critical. Ref. [22] note that research on the cognitive effect of subtitles clarifies why viewers gain or lose comprehension under varied subtitle conditions. They also warn that most studies rely on short videos with few subtitle events, a design that ignores the cumulative influence of extended exposure and highlights the need to balance ecological validity with experimental rigor.

The subtitling landscape has changed through automation technologies. Ref. [23] labels the earliest output as “verbatim subtitles”, transcripts produced by speech-to-text software and aligned with dialog. Today, sophisticated neural systems generate subtitles far faster and at lower cost, yet serious shortcomings persist in recognition accuracy, line segmentation, and reading speed [6]. Automated subtitles often flash too quickly and repeat dialog word for word, which overwhelms viewers and harms comprehension.

Viewer studies report persistent skepticism toward machine output. Ref. [24] finds that audiences question the quality of machine-translated subtitles. Ref. [25] likewise observe that viewers accept machine subtitles for access but face a higher cognitive load than with human translations, as shown by longer fixations and additional rereads. These effects stem from faults in speech recognition, translation, and presentation speed, and resemble [26] idea of “subtitling blindness”, a state in which attention to text reduces engagement with on-screen images.

The challenges multiply when subtitling crosses writing systems. Arabic–English translation illustrates these complexities because viewers must navigate linguistic differences and disparities in visual processing. Ref. [4] showed that the perceptual span, the window of effective vision during reading, depends on direction. For Arabic readers, “performance was superior when windows extended leftward […] essentially the reverse of […] English.” This directional asymmetry poses specific cognitive challenges for Arabic audiences, particularly when they shift between right-to-left script and left to right visual scanning patterns common in Western cinematography. While our subtitle areas of interest (AOI) followed standard bottom-center placement with right-aligned Arabic text, Arabic readers’ rightward-to-leftward processing pattern may create unique attentional dynamics that warrant consideration in cross-script subtitle research. The right-to-left text alignment within the centered subtitle area may interact with viewers’ natural reading patterns in ways that could influence gaze distribution between subtitle and picture areas.

The arrival of LLMs marks the latest phase of automation and promises contextually rich translations, yet whether these advances reduce cognitive burden remains unclear. LLMs can produce fluent sentences but still violate subtle subtitle conventions. Ref. [27] states that subtitling’s “distinctive meaning potential” allows it to serve “not only as a translation method but as a form of intercultural mediation.” Such mediation calls for cultural competence that AI systems still lack, especially for content that involves cultural references, idiomatic expressions, metaphor, emotional nuance, and humor based on wordplay, elements that recent work lists among the most frequent errors in LLM output.

Web-based eye tracking appears to be an increasingly valuable tool for subtitle research, potentially offering both methodological rigor and practical advantages. Building on this perspective, Ref. [28] note that while dynamic systems using wearable devices allow participants to interact with their environment, static or remote systems, particularly those using fixed monitors, continue to dominate in research settings due to their capacity for controlled stimulus presentation. This is especially pertinent to our study, which employs web-based remote eye tracking to measure gaze patterns on subtitle areas while ensuring experimental consistency. Advances in this technology have made remote measurements comparable to those obtained in lab settings, with metrics from online studies closely matching those from dedicated hardware [29,30,31]. The remote approach also reduces costs, facilitates access to geographically diverse samples, and enables participants to view media in familiar environments. As a result, researchers can now recruit larger participant pools, such as 150 individuals in recent studies compared to the 15 to 30 typically used in earlier work, which is a notable strength of contemporary web-based eye-tracking research [32].

Overall, automatic subtitle use, the cognitive demands of multimodal integration, cross-script processing challenges, and the rise of automated translation create an urgent need for empirical investigation. As audiences encounter LLM-generated subtitles across platforms, evidence about their cognitive effects becomes essential for policy on media accessibility and translation standards. The present study meets this need by applying eye tracking to compare cognitive load under human and GPT-4o Arabic subtitles and clarifies whether gains in technological efficiency raise viewer cognitive burden.

## 3. Methods

This study examined how the subtitle generation method affects the cognitive load of Arabic viewers by means of a web-based eye-tracking platform. Using a between-subjects experimental design, we compared professionally produced human subtitles with those generated by GPT-4o and treated participants’ English proficiency as a moderating factor. The eye-tracking setup captured, in real time, the distribution of visual attention between subtitle and picture areas and thus yielded quantitative indices of cognitive processing during naturalistic viewing.

### 3.1. Participants

The sample comprised 82 native Arabic speakers with normal or corrected-to-normal vision (46 females, 36 males) whose ages ranged from 18 to 50 years (*M* = 29.4, *SD* = 7.2). Recruitment proceeded through university networks, community organizations, and social media. Each participant completed a screening questionnaire that confirmed native proficiency in Arabic and daily use of the language. A priori power analysis (*α* = 0.05, power = 0.80, estimated effect size *d* = 0.65) indicated that 38 participants per condition were required, and we set the recruitment target at 45 to offset potential data loss in remote eye tracking.

We initially recruited 107 participants (57 were allocated to the human-created subtitle condition; 50 to the GPT-4o condition). After quality control, 25 datasets were excluded when the software rated data quality as Very Low (sampling rate 0 Hz with no usable fixations) or Low (sampling rate ≥ 1 Hz with fewer than 50% fixations across the viewing period). The final sample included 41 participants in each condition. English proficiency was assessed with an adapted version of the Cambridge General English Test (score range 0–25). Participants were classified as Basic (0–8, *n* = 14), Intermediate (9–16, *n* = 32), or Advanced (17–25, *n* = 36). Ethical clearance was granted by the Kent State University Institutional Review Board (Application 1664), and informed consent was obtained from all participants.

### 3.2. Design

We implemented a between-subjects design with subtitle type (professional human versus GPT-4o Arabic subtitles) as the independent variable and English proficiency as a moderator. The dependent variables were eye-tracking metrics that index cognitive load, namely fixation count, mean fixation duration, total fixation time, gaze distribution (proportion of time on subtitles versus visual content), and K-coefficient (attention concentration).

A between-subjects approach prevented carryover and learning effects that could arise in repeated viewings. Participants were randomly assigned to one subtitle condition, and stratification by proficiency ensured balanced groups.

### 3.3. Materials

The experimental stimulus was Episode 3, Syria, from Season 1 of State of the Union, a ten-minute episode that presents a dialog between a couple in a London pub before their weekly marriage counseling session. The episode was chosen for its controlled visual composition (mainly medium close-ups), high linguistic density (about 138 words per minute), and narrative richness. Forty-three percent of the dialog involves cultural references, idioms, metaphors, emotional nuance, or wordplay, features that present translation challenges.

Both subtitle conditions followed identical technical specifications so that the generation method remained the sole independent factor; details appear in Table 1. For the GPT-4o condition, Arabic subtitles were produced with GPT-4o (March 2025) through TransVisio, a platform that submits prompts and retrieves SubRip Text files via API [33]. The prompt specified timing, technical settings, and cultural adaptation. No post-editing occurred, which preserved the integrity of the LLM output and allowed a direct comparison with human translations. The human-created subtitle condition used the Modern Standard Arabic (MSA) track from Amazon Prime, created by professional subtitlers in line with industry standards.

The study used RealEye (https://www.realeye.io, accessed on 1 March 2025), a web-based eye-tracking platform that records gaze through standard webcams and thus collects data in participants’ usual viewing environments while still allowing experimental control. The system samples at 30 Hz and relies on a real-time gaze prediction algorithm that reaches an average accuracy of 1.5–2.0 degrees of visual angle [34]. Key acquisition and filtering parameters are detailed in Table 2.

Our definition of the subtitle area followed established methodological practices. Ref. [17] similarly described the region as “deliberately larger than the actual area that displayed the subtitles to account for small vertical and horizontal inaccuracies in the recording of the eye movements,” which confirms our approach to AOI specification.

Eye tracking provides a well-established method for examining how viewers distribute visual attention during subtitle reception, supplying quantitative evidence about gaze behavior through analysis of eye fixations relative to caption areas during intervals when captions appear on screen. This approach yields measures such as fixation location and duration, which serve as direct indicators of visual attention during multimodal processing. Central to this methodology is the definition of areas of interest (AOIs), regions of the frame selected for detailed analysis, with subtitle research typically specifying two main AOIs: the area that presents subtitles and the remaining area that carries pictorial information. This arrangement permits systematic comparison of attention allocation between textual and visual elements, enabling researchers to understand how viewers navigate between reading subtitles and processing visual content.

To describe attention distribution between subtitle and picture areas, we extracted a comprehensive set of eye-movement metrics. Fixations, brief pauses during which the eye gathers information, formed the basis of analysis. Four primary indices followed: fixation count, the frequency of pauses within an AOI; mean fixation duration, the average length of each pause that reflects processing intensity; total fixation time (dwell time), the cumulative duration of all pauses within an AOI; and the K-coefficient, a ratio that combines fixation duration with the amplitude of the subsequent saccade to indicate the degree of attention concentration. Operational definitions and interpretive guidelines for all five metrics appear in Table 3.

These metrics together should reveal how viewers allocate limited cognitive resources while processing multimodal information and therefore provide a basis for comparing different subtitle-generation methods and their associated cognitive demands.

### 3.4. Procedure

Prior to the experiment, each participant completed electronic informed consent, a brief background questionnaire, and the Adapted English Proficiency Test, a sequence that required five to seven minutes. The system then verified that the participant’s equipment met minimum standards: a webcam with at least 720 p resolution, a stable internet connection of 5 Mbps or higher, and adequate front lighting. Next, RealEye ran its automated 39-point calibration; participants sat about 60 cm from the screen and kept head movement to a minimum, and the software repeated calibration automatically when precision thresholds were not met.

Participants were randomly assigned to view the episode with either professional human subtitles or GPT-4o Arabic subtitles and received instructions to watch naturally, as they normally would. To preserve ecological validity, participants also answered a short comprehension quiz and completed a survey about the episode; those results fall outside the scope of this article, though comprehension outcomes favored human-translated subtitles, with participants achieving higher accuracy rates across factual recall, cultural reference recognition, and overall narrative coherence. These comprehension differences reinforced that the increased cognitive load observed with GPT-4o subtitles was associated with reduced understanding effectiveness. Two AOIs framed gaze analysis: Subtitle AOI, a strip at the bottom of the frame (16% X, 84% Y; 63% width × 16% height), and Picture AOI, the remaining screen space. Both regions stayed active for the full video (554.28 s), which allowed uninterrupted observation of attention distribution.

RealEye captured gaze at 30 Hz during viewing and registered the proportion of fixations within each Area of Interest. The processing pipeline extracted raw coordinates, applied the I-VT filter to identify fixations, mapped each fixation to an AOI, and derived the eye-tracking indices used to compare subtitle conditions. We compared group differences with independent-samples *t* tests and entered English proficiency as a factor in a two-way analyses of variance (ANOVA) to examine moderation. All tests report effect sizes and 95% confidence intervals, and a Bonferroni correction adjusted the *α* level for multiple comparisons. Statistical procedures were carried out in Python 3.13.2.

## 4. Results

The dependent measures were eye-tracking indices of cognitive load during subtitle reception. These indices comprised fixation count, mean fixation duration, total fixation time, and the proportion of gaze directed to subtitle versus visual narrative areas. We also examined K-coefficient values for both subtitle and picture regions to quantify patterns of attention concentration. These measures enabled direct comparison of subtitle conditions and assessment of how English proficiency moderated any differences.

### 4.1. Eye-Tracking Indicators of Cognitive Load

After we verified the assumptions required for parametric analysis, we used independent samples *t* tests to analyze the primary metrics (see Table 4).

**Figure 1:**
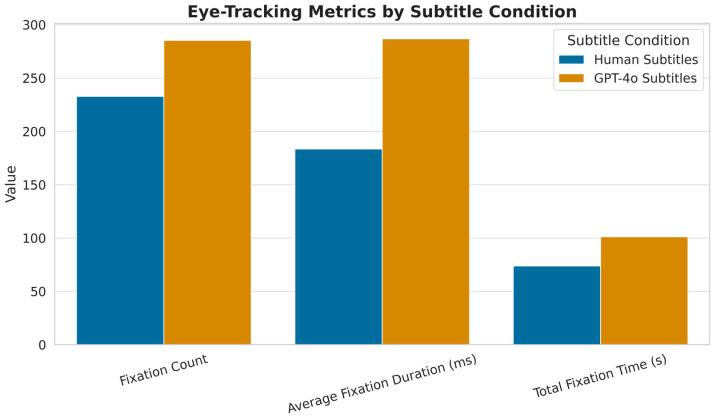
Eye-Tracking Metrics by Subtitle Condition.

Participants in the GPT-4o condition recorded significantly more fixations (*M* = 285.42, *SD* = 170.53) than those in the human condition (*M* = 232.84, *SD* = 157.00), *t*(80) = 2.78, *p* = 0.013, *d* = 0.39; this small-to-medium effect shows that viewers revisited the subtitle area more often with GPT-4o translations. Mean fixation duration displayed an even larger gap, with GPT-4o viewers pausing longer (*M* = 286.91 ms, *SD* = 71.66) than their counterparts (*M* = 183.51 ms, *SD* = 67.29), *t*(80) = 3.61, *p* = 0.001, *d* = 1.74, evidence of higher processing effort. Total fixation time followed the same trend; participants looked at subtitles longer in the GPT-4o condition (*M* = 101.29 s, *SD* = 58.47) than in the human condition (*M* = 73.80 s, *SD* = 60.22), *t*(80) = 2.82, *p* = 0.006, *d* = 0.60. Taken together, these indicators confirm that GPT-4o subtitles impose greater cognitive load than professional translations. This pattern is visualized in Figure 1, which presents each of the three fixation-based measures side by side, highlighting the consistent elevation in viewer effort under the GPT-4o condition.

Additionally, when considering total fixation time, participants spent significantly more time viewing subtitles in the GPT-4o condition (*M* = 101.29 s, *SD* = 58.47) than in the human condition (*M* = 73.80 s, *SD* = 60.22), *t*(80) = 2.82, *p* = 0.006, *d* = 0.60. Consequently, this result further supports the indication of greater overall cognitive load associated with the machine-generated subtitles. The summary in Figure 1 reinforces this interpretation, showing not only longer durations but also a higher frequency and total time of subtitle engagement when viewers were exposed to GPT-4o output.

### 4.2. AOI Fixation Distribution Analysis

We examined how viewers divided visual attention between subtitle and narrative areas in the two experimental conditions, and Table 5 lists the complete set of fixation and gaze metrics for the two defined AOIs.

Viewers in the human-created subtitle group directed 20.6% of their fixations to the subtitle strip, whereas those in the GPT-4o group devoted 24.4%. This represents an 18.4% rise in attention to text with the LLM subtitles; the difference reached significance, *t*(80) = 2.36, *p* = 0.021, *d* = 0.52. These findings gain context alongside professional subtitling standards. Ref. [35] state that subtitles ought to be an unobtrusive, ancillary translation that does not excessively attract attention, either formally or linguistically. Standard practice also limits interlingual subtitles to two lines that cover no more than two-twelfths of the screen area [35], a constraint respected in both conditions. Since layout remained constant, the extra attention drawn by GPT-4o subtitles likely reflects their language choices rather than their placement.

Gaze-duration data revealed a similar pattern. Participants who watched GPT-4o subtitles spent far more time on subtitle text (*M* = 80,397 ms, *SD* = 58,392) than those who watched professional subtitles (*M* = 44,304 ms, *SD* = 62,375), *t*(80) = 2.82, *p* = 0.006, *d* = 0.67, an 81.5% increase that implies a large shift in attention away from pictorial information. Time to first fixation on subtitles did not differ between conditions (human: *M* = 16,012 ms, *SD* = 12,624; GPT-4o: *M* = 7074 ms, *SD* = 10,879), *t*(80) = 0.95, *p* = 0.346, *d* = 0.21, which suggests similar early orientation across groups. The subtitle K-coefficient rose from 0.10 to 0.30, *t*(80) = 2.85, *p* = 0.006, *d* = 0.63, indicating longer fixations paired with shorter saccades and therefore deeper cognitive processing when viewers read GPT-4o subtitles; in contrast, the corresponding picture-area K-coefficient increase from 0.10 to 0.18 did not reach significance, *t*(80) = 1.51, *p* = 0.137, *d* = 0.33.

### 4.3. English Proficiency as Moderator

To examine whether English proficiency moderated the effect of subtitle condition on attention patterns, we conducted ANOVAs with subtitle condition and proficiency level as factors, and Table 6 lists the primary AOI metrics across proficiency levels and conditions.

**Figure 2:**
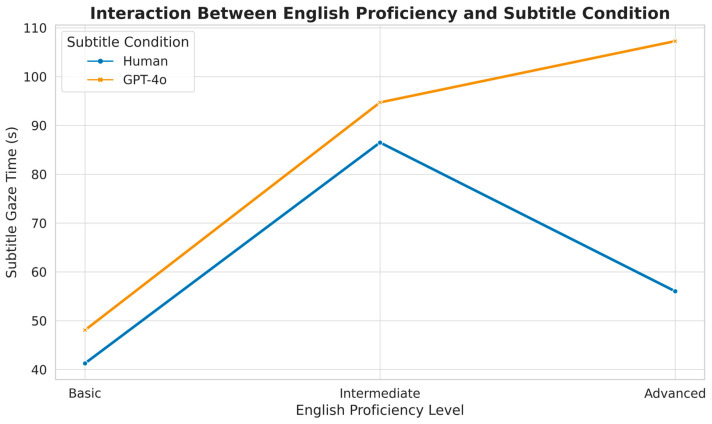
Interaction Between English Proficiency and Subtitle Condition.

A significant interaction emerged for time to first fixation (TTFF), *F* (2, 76) = 7.84, *p* < 0.001, partial η^2^ = 0.171. Within the GPT-4o group, TTFF decreased as proficiency rose; advanced viewers located the subtitle area more quickly (*M* = 4.9 s, *SD* = 1.90) than basic viewers (*M* = 15.0 s, *SD* = 3.90), *t*(23) = 8.76, *p* < 0.001, *d* = 3.27. Gaze time metrics displayed a comparable pattern. A two-way ANOVA returned significant interactions for subtitle gaze time, *F* (2, 76) = 9.37, *p* < 0.001, partial η^2^ = 0.198, and picture area gaze time, *F* (2, 76) = 6.82, *p* = 0.002, partial η^2^ = 0.152. Advanced participants spent more time on subtitles in the GPT-4o condition (26.7% of total viewing time) than in the human subtitle condition (20.8%), *t*(34) = 7.25, *p* < 0.001, *d* = 2.42. Figure 2 visualizes this interaction, showing a clear increase in subtitle gaze time among advanced viewers, particularly under the GPT-4o condition.

Subtitle gaze time increased significantly with proficiency in the GPT-4o group, suggesting heightened sensitivity to nuanced language flaws in machine-generated output.

**Figure 3:**
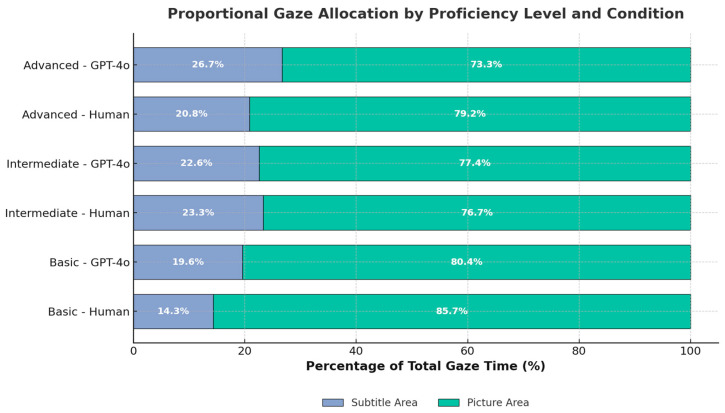
Proportional Gaze Allocation by Proficiency Level and Condition.

Figure 3 complements this analysis by depicting proportional gaze allocation across subtitle and picture areas by proficiency level. This view reveals that advanced users directed a larger share of visual attention to subtitles under the GPT-4o condition, shifting cognitive resources away from the narrative track. This gaze redistribution pattern implies deeper engagement with and increased cognitive scrutiny of AI-generated subtitles among higher proficiency viewers.

As shown in Figure 3, higher proficiency viewers in the GPT-4o condition devoted more relative gaze time to subtitles than to the picture area, reflecting intensified linguistic processing.

### 4.4. Summary of Key Findings

The eye-tracking analysis revealed consistent patterns that indicate distinct cognitive demands across subtitle conditions. Subtitles produced by GPT-4o yielded significantly higher scores on every cognitive-load metric than professional human translations (*p* < 0.05). Furthermore, viewers in the GPT-4o condition directed more attention to the subtitle strip than to visual narrative content (*d* = 0.52), evidence of greater resource allocation to text. K-coefficient values supported this view, because GPT-4o subtitles elicited more concentrated attention, with longer fixations and shorter saccades, a profile that shows increased cognitive effort when viewers read subtitles. English proficiency also moderated these effects. Advanced participants presented the largest shifts in gaze distribution between conditions (partial η^2^ = 0.198), which suggests that those with stronger language skills proved more sensitive and adaptive to subtitle quality. Overall, the findings show how the subtitle generation method guides viewers’ cognitive resource use during audiovisual engagement, especially in cross-script contexts that call for rapid alternation between writing systems.

## 5. Discussion

### 5.1. Hypothesis Testing and Support

Subtitles generated by GPT-4o imposed greater cognitive load than professional human translations. Fixation counts rose, mean fixation duration lengthened, and total fixation time increased in the GPT-4o condition; together these metrics show that reading machine subtitles consumed more mental resources. The size of these effects, especially the marked rise in mean fixation duration, indicates that GPT-4o output required greater effort than expert translations, even though the model possesses advanced language capabilities.

The data also confirmed the prediction concerning attention allocation. Viewers who watched GPT-4o subtitles directed a larger share of fixations toward the subtitle band than those who watched human translations. This shift in attention from narrative images to text implies extra cognitive work when viewers extract meaning from LLM output and thus lower engagement with visual narrative elements. A 200 percent increase in K-coefficient values within the subtitle area reinforces this interpretation and signals a change from ambient scan patterns to focal analysis, a profile typical of elevated cognitive demand.

English proficiency moderated these outcomes as anticipated. Participants with advanced proficiency displayed the largest between-condition differences and spent far more time on GPT-4o subtitles than on human ones. Significant interaction effects for time spent on subtitles and on picture areas confirm that higher linguistic competence intensifies sensitivity to subtitle quality. Paradoxically, viewers with greater language resources experienced stronger disruption from GPT-4o translations; the capacity to detect subtle deficiencies appears to raise cognitive burden rather than reduce it.

The inferred cognitive load increases with GPT-4o subtitles, although surface-level fluent, since they impede processing fluency—”the experienced ease with which a mental operation is performed” [36]. Fluency of processing influences various cognitive judgments, including truthfulness judgments, in which information that is easier to process is rated as more trustworthy [37,38,39] as cited in [40]. In audiovisual translation, successful subtitles create what can be termed as a “fluency illusion,” where well-designed translations result in natural flow in language and readability so that subtitles become “the exact words uttered by the actors, exceeding their role as simple translations” [41]. However, machine-translated errors can “break [the] illusion and momentarily disrupt the immersive experience” [42].

Therefore, our findings show that GPT-4o subtitles created a particular problematic scenario; they possessed sufficient surface-level fluency to support further reading interaction, but contained persistent meaning distortions, context loss, and cultural adaptation failures that violated processing expectations. This surface-level fluency initially increased viewer trust, paradoxically leading to closer scrutiny rather than dismissal. Unlike obviously flawed traditional MT that viewers might ignore, the near-native quality compelled viewers to invest cognitive effort reconciling conflicting information. This gap between forecasted fluency and achieved semantic adequacy drove viewers towards effortful reconciliation processes, the cause of the longer fixation durations, increased re-reading behaviors, and larger K-coefficient values in the GPT-4o condition.

To demonstrate the types of errors that most likely led to the observed cognitive processing difficulties, Table 7 provides some examples of these systematic translation failures.

These examples shown in Table 7 account for why GPT-4o subtitles imposed greater cognitive effort despite surface-level fluency. Cultural references like ‘pint’ and ‘dry roasted’ invited audiences to bridge in the disconnect between literal translations and visual context. Mistranslations like ‘effete’ → ‘nice’ demanded reconciling cognitively whenever subtitle meaning was at odds with situational cues. Most egregiously, complete semantic failures in processing sexual content and literary allusions most likely provoked successive re-reading and effortful meaning-construction processes. These error patterns explain our 56% increase in fixation duration and tripled K-coefficient values observed across our eye-tracking data, as viewers engaged in the additional cognitive work of parsing, questioning, and reinterpreting problematic subtitle content.

### 5.2. Comparison with Previous Research

Our findings align with and extend existing research on automated subtitle processing. Similar patterns appear in [43], who reported greater mental effort with raw machine translations, and in [44], who recorded higher extraneous load with automatic subtitles. The larger effect sizes observed in our study show that subtitles produced by GPT-4o demand more cognitive resources than those created with traditional MT, so recent advances in language modeling do not ease the problem.

Attention data reveal this effect. In the GPT-4o condition, viewers spent more time looking at the subtitles than those viewing human translations. This difference from [24] suggests participants altered their viewing method. Traditional systems contained clear mistakes that viewers ignored at once, but the near-fluent rendering by GPT-4o prompted them to read each line with greater care. Ref. [25] maintain that doubts about MT drive viewers to examine subtitles more closely, which our results confirm. In addition, the K-coefficient in the subtitle region rose from 0.10 to 0.30, indicating a shift from peripheral to focused reading. This outcome contrasts with the minimal attention that effective subtitles aim to yield.

While our primary focus was on measuring cognitive load, it is likely that translation errors in GPT-4o’s subtitles contributed substantially to the observed processing difficulties. Although a full error analysis was beyond the study’s scope, an initial review showed recurring issues, such as meaning distortions, missing contextual cues, and failures in adapting culturally specific content. Grammatical mistakes in Arabic (e.g., gender and number agreement) were also common. These errors likely forced viewers to exert more effort to infer intended meanings, which may help explain the rise in fixation durations and K-coefficient values. Some errors appeared more cognitively demanding than others, depending on the interpretive work they required. Roughly 43% of the dialog included idioms, metaphors, emotional nuance, cultural references, or wordplay, elements known to challenge automated translation. Inaccurate renderings of these segments possibly created uneven processing demands. Also, cultural and pragmatic errors seem to require more contextual reasoning than straightforward lexical errors. Therefore, this may account for the fact that participants with stronger English proficiency, those most attuned to such subtleties, showed the largest increases in cognitive load. Future work would benefit from a systematic classification of subtitle error types and their corresponding eye-tracking signatures.

Furthermore, participants devoted between 20% and 25% of their viewing duration to subtitles, remaining under the 30% ceiling set by [45], yet text quality determined their focus. A normal share of viewing time did not produce a standard viewing pattern. Professional subtitlers shorten text and remove redundancy so imagery can convey the plot [46]. Our finding of an 81.5% rise in subtitle attention shows that GPT-4o output upset this balance. Ref. [6] warned that automated captions can overload viewers; our data point to a new issue. Minor errors in LLM output forced viewers to parse each subtitle carefully. Instead of aiding comprehension, GPT-4o subtitles vied with the visual content for mental effort.

### 5.3. Study Validity and Limitations

Several factors reinforce the validity of these findings. The between-subjects design removed potential carry-over effects, and random assignment with stratification kept group composition balanced. Identical technical specifications across conditions isolated the subtitle generation method as the only manipulated variable. This methodology reflects recent advances in web-based eye tracking, especially studies that use RealEye for subtitle research. The authors of [32], for instance, assessed swearword translation with RealEye and 150 participants across five conditions; their work, like ours, collected data in natural settings while preserving experimental control. The close correspondence between our fixation count, mean fixation duration, and total fixation time and theirs, all recorded at 30 Hz, validates our approach. Convergence of results across studies that apply the same technology to distinct translation issues strengthens confidence in web-based eye tracking as a reliable method for AVT research.

Some limitations temper these conclusions. The single ten-minute episode, although useful for content control, restricts generalization across genres and viewing duration. Longer sessions may reveal cumulative fatigue that enlarges condition differences; most current eye tracking research employs short clips, so it may underestimate strain from extended subtitle work. The sample also drew mostly from university-affiliated viewers and thus likely overrepresents higher educational backgrounds and familiarity with subtitles. Subtitle automaticity may vary with experience, so the findings may not extend to less experienced audiences. Finally, the 30 Hz sampling rate of the web platform, though adequate for fixation measures, trails laboratory hardware and may miss very brief fixations. More specifically, while this sampling rate proves sufficient for capturing fixation patterns, it may have underestimated rapid saccadic movements and micro-fixations that could reveal additional cognitive processing differences. Laboratory-grade eye trackers operating at 250–1000 Hz might detect brief corrective saccades or rapid re-fixations that the RealEye system missed. Moreover, agreement between our results and both laboratory reports and other RealEye studies that address diverse subtitle issues suggests that this technical limit did not alter the observed cognitive load gap between human and machine translations.

### 5.4. Future Research Directions

Our findings suggest several lines of inquiry that could advance automated subtitle research. Longitudinal studies that examine whether extended exposure to LLM generated subtitles produces adaptation effects or cumulative fatigue would clarify long term viewer outcomes, while cross linguistic work that compares script combinations such as Chinese-English or Spanish-Arabic could reveal whether the cognitive load patterns reported here are specific to Arabic or reflect broader challenges in automated translation across distinct writing systems. The pronounced proficiency effects observed in this study underscore the need for adaptive subtitle systems that tailor translation strategies to viewer characteristics; particularly promising would-be dynamic systems that adjust subtitle complexity, presentation speed, or translation approach in real-time based on user proficiency levels or ongoing engagement metrics. Such adaptive technologies could monitor eye-tracking patterns, reading speeds, or comprehension indicators to automatically optimize subtitle delivery for individual viewers, potentially reducing cognitive load while maintaining accessibility. Furthermore, eye tracking data could inform real-time quality metrics that allow LLMs to optimize subtitles for cognitive efficiency rather than linguistic accuracy alone. A near-term priority involves the identification of linguistic features that raise cognitive load, especially cultural references, idiomatic expressions, and humor, items that proved most troublesome for automated systems in our materials.

In addition, the web-based eye-tracking method employed in this research is found to possess strong adaptability in the measurement of different types of AI-generated content and presentation formats. The approach can be extended to study the cognitive impact of several other conditions like AI-generated video content containing differently placed banners or overlays, real vs. AI-generated image comparisons within multimedia environments, and other newly emerging types of automated content creation. The attention metrics and cognitive load indicators measured through eye tracking provide a strong basis for understanding how different AI-generated features affect viewer processing across diverse media formats.

### 5.5. Study Contributions

This study offers several contributions to knowledge of automated subtitle processing in the era of large language models. It presents one of the first systematic eye tracking comparisons between professional Arabic subtitles and those generated by GPT-4o, showing that advanced AI introduces measurable cognitive costs for viewers. Although these models create translations that appear fluent, they still raise processing demands, a tension that challenges media accessibility because speed gains may carry a cognitive price. English proficiency emerged as a key moderating factor; viewers most able to spot quality differences, including language learners who depend on subtitles, faced the greatest disruption. As streaming services increase their use of automated subtitling, these findings support continued human oversight in AVT, especially for content that crosses distinct writing systems and already taxes cognitive resources. Given the anticipated widespread adoption of LLM-based subtitle systems across commercial and fan streaming platforms, as well as other media distribution channels, these cognitive load findings carry significant implications for accessibility policies and industry quality standards and underscore the need to balance automation efficiency with viewer cognitive burden in future subtitle deployment strategies.

Finally, the finding that participants with lower English proficiency were less attuned to quality variation between human and GPT-4o Arabic subtitles has broader implications for the development of Arabic in the digital age. It is assumed that as more subtitles will be generated by LLMs and deployed across streaming platforms and educational content, less competent source-language viewers, who represent a large percentage of subtitle-dependent audiences, will be less discerning of machine-generated linguistic patterns and cultural adaptations. Therefore, this reduced sensitivity would make the gradual incorporation of AI-generated Arabic expressions, syntactic structures, and cultural framings within audiences’ own linguistic repertoires feasible, particularly by young speakers whose language acquisition occurs simultaneously with ubiquitous exposure to automated translation systems.

## Figures and Tables

**Table 1:** Subtitle Design Parameters.

	Specification	Justification
Maximum Characters/Line	42	Aligns with video-on-demand (VOD) platform limits (such as Amazon Prime and Netflix) for single-byte languages including Arabic
Maximum Lines/Unit	2	Reflects standard convention for subtitle segmentation and viewer comfort
Reading Speed	20 characters/s	Upper-end industry norm; used in studies and supported by viewer processing capacity at high speeds
Display Duration	1–6 s	Flexible to match speech rate and subtitle length; within industry norms
Font	Traditional Arabic	Tested for legibility; shows strong character distinction and clarity for Arabic script
Font Size	35 points	Based on visual ergonomics for screen reading at typical desktop and laptop viewing distances
Position	Bottom-center	Industry standard to minimize visual distraction
Text Alignment	Right aligned	Necessary for natural Arabic reading flow and script orientation

**Table 2:** RealEye System Configuration.

	Specification	Function
Sampling Rate	30 Hz	Temporal resolution
Calibration Grid	39-point automated	Spatial accuracy
Fixation Filter	*I-VT* (Velocity Threshold Identification)	Gaze classification
Minimum Fixation	80–400 milliseconds	Processing threshold
Noise Reduction	200 ms window	Signal cleaning
Velocity Threshold	150°/second	Saccade identification

**Table 3:** Operational Definitions and Cognitive Interpretations of Eye-Movement Metrics.

Metric	Operational Definition	Units	Cognitive Interpretation
Fixation count	Number of discrete fixations recorded within the subtitle area of interest (AOI) across the clip	Count	Higher values indicate denser scanning or re-reading of subtitle text
Mean fixation duration	Sum of individual fixation durations divided by the number of fixations in the subtitle AOI	Milliseconds	Longer averages reflect deeper lexical/semantic processing of the subtitle line
Dwell time (total visit duration)	Total time, fixations + intra-AOI saccades, spent in the subtitle AOI	Seconds (and/or % of clip time)	Greater dwell signals more attentional investment in reading subtitles, potentially at the expense of watching the image track
Gaze distribution	Proportion of all fixations (or dwell time) allocated to each predefined AOI (e.g., subtitles, speaker face, background)	Proportion	A skewed distribution toward subtitles shows a strong attentional pull; a balanced spread implies joint processing of text and image
K-coefficient	Standardized difference between fixation duration (*FD*) and saccade amplitude (*SA*)	Dimensionless	+*K* ≈ focal, detail-oriented viewing; −*K* ≈ ambient, exploratory scanning

Note. *FD* = fixation duration; *SA* = saccade amplitude. Positive *K* values indicate concentrated visual attention (long fixations paired with short saccades), whereas negative values reflect a more exploratory scanning mode.

**Table 4:** Fixation Metrics by Subtitle Condition.

Metric	Human Condition *M* (*SD*)	GPT-4o Condition *M* (*SD*)	*t*(80)	*p*	*d*
Fixation Count	232.84 (157.00)	285.42 (170.53)	2.78	0.013 *	0.39
Average Fixation Duration (ms)	183.51 (67.29)	286.91 (71.66)	3.61	0.001 **	1.74
Total Fixation Time (s)	73.80 (60.22)	101.29 (58.47)	2.82	0.006 **	0.60

Note. *p* < 0.05 *, *p* < 0.01 **.

**Table 5:** Comprehensive AOI Metrics Comparison by Condition.

Metric	Human Subtitles	GPT-4o Subtitles	% Change	Statistical Significance
Fixation Count (Picture Area)	28,866 (79.4%)	34,326 (75.6%)	+18.9%	*U* = 723, *p* = 0.278, *d* = 0.24
Fixation Count (Subtitle Area)	7481 (20.6%)	11,104 (24.4%)	+48.4%	*t*(80) = 2.36, *p* = 0.021, *d* = 0.52
Avg. Fixation Duration (Picture Area)	198 ms	257 ms	+29.8%	*t*(80) = 3.51, *p* < 0.001, *d* = 0.77
Avg. Fixation Duration (Subtitle Area)	197 ms	266 ms	+35.0%	*t*(80) = 3.91, *p* < 0.001, *d* = 0.86
Total Time Spent (Picture Area)	164,466 ms	225,651 ms	+37.2%	*U* = 620, *p* = 0.041, *d* = 0.46
Total Time Spent (Subtitle Area)	44,304 ms	80,397 ms	+81.5%	*t*(80) = 2.82, *p* = 0.006, *d* = 0.67
Time to First Fixation (Picture Area)	1042 ms	532 ms	−48.9%	*U* = 1 121, *p* = 0.008, *d* = 0.60
Time to First Fixation (Subtitle Area)	16,012 ms	7074 ms	−55.8%	*t*(80) = 0.95, *p* = 0.346, *d* = 0.21
K-Coefficient (Picture Area)	0.10	0.18	+80.0%	*t*(80) = 1.51, *p* = 0.137, *d* = 0.33
K-Coefficient (Subtitle Area)	0.10	0.30	+200.0%	*t*(80) = 2.85, *p* = 0.006, *d* = 0.63

Note. All tests are Welch two-sample t-tests except skewed or count data, which used Mann–Whitney U after log transformation.

**Table 6:** Eye-Tracking Metrics by Proficiency Level and Condition.

Proficiency Level	Condition	Time to First Fixation (s)	Subtitle Gaze Time (s)	Picture Area Gaze Time (s)
Basic	Human	19.49 (*SD* = 4.75)	41.26 (*SD* = 14.20)	247.12 (*SD* = 42.70)
	GPT-4o	15.02 (*SD* = 3.90)	48.07 (*SD* = 16.80)	197.30 (*SD* = 38.50)
Intermediate	Human	12.35 (*SD* = 3.10)	86.50 (*SD* = 21.50)	285.16 (*SD* = 53.60)
	GPT-4o	7.28 (*SD* = 2.80)	94.72 (*SD* = 23.20)	325.02 (*SD* = 61.90)
Advanced	Human	15.50 (*SD* = 4.20)	56.03 (*SD* = 17.60)	213.15 (*SD* = 47.30)
	GPT-4o	4.92 (*SD* = 1.90)	107.27 (*SD* = 25.40)	294.27 (*SD* = 57.80)

Note. Time measures are in seconds, with standard deviations in parentheses. Basic (*n* = 14), Intermediate (*n* = 32), and Advanced (*n* = 36) groups were distributed across subtitle conditions.

**Table 7:** Translation Errors Contributing to Cognitive Load.

English Source	Human Arabic Translation	GPT-4o Arabic Translation	Back Translation of GPT-4o	Error Analysis	Error Categorization
Pint of London Pride and a pack of dry roasted, please.	أريد كوباً من الجعة وكيساً من المكسرات من فضلك	كبيرة لندن برايد ورزمة فول سوداني، لو سمحت	Large London Pride and a bundle of peanuts, please	GPT-4o literally translates “pint” as “large” and fails to understand British pub culture context	Cultural Adaptation + Literal Translation
The ‘ooh’ sounds a bit, you know, effete.	هذا الصوت يبدو واهناً	أوه تبدو قليلًا، كما تعلم، لطيف	Ooh sounds a little, you know, nice	Effete (meaning weak/unmanly) completely mistranslated as “nice”—opposite meaning	Critical Mistranslation
A lot of men would like to hear their wives use the phrase ‘fuck each other stupid’.	رجال كثر يرغبون في سماع زوجاتهم يستخدمن عبارة، “لنقم علاقةً رائعةً”	الكثير من الرجال يحبون أن يسمعوا زوجاتهم يستخدمون عبارة “اللعنة، توقفوا عن الغباء”	A lot of men like to hear their wives use the phrase ‘damn, stop being stupid’	Sexual/intimate language completely misunderstood and mistranslated	Sexual Content Mishandling + Complete Mistranslation
Do not go gentle into that good night.	لا تستسلم بلطف للرحيل	لا تستسلم بسهولة لتلك الليلة الجميلة	Do not surrender easily to that beautiful night	Famous Dylan Thomas quote rendered literally, missing poetic/metaphorical meaning about death/aging	Literary Reference + Metaphorical Failure

## Data Availability

The datasets generated and/or analyzed during this study are not publicly available due to data privacy concerns related to the collection of information as part of a continuous PhD project, with the dissertation defense yet to occur and the data representing only a portion of the full project. However, the data are available from the corresponding author on reasonable request.
